# Development of an in-house quantitative ELISA for the evaluation of different Covid-19 vaccines in humans

**DOI:** 10.1038/s41598-022-15378-1

**Published:** 2022-07-04

**Authors:** Mariem Gdoura, Fatma Ben Ghaloum, Meriem Ben Hamida, Wafa Chamsa, Henda Triki, Chokri Bahloul

**Affiliations:** 1 Laboratory of Clinical Virology, Institut Pasteur de Tunis, Tunis, Tunisia; 2 Research Laboratory « Virus, Vector and Hosts » LR20IPT02, Tunis, Tunisia; 3 Faculty of Pharmacy of Monastir, University of Monastir, Monastir, Tunisia; 4 Vaccinologie et Développement Biotechnologique, LR11IPT01 Microbiologie Moléculaire, Institut Pasteur de Tunis, Université de Tunis El Manar, 13, Place Pasteur, BP 74, 1002 Tunis-Belvedere, Tunisia

**Keywords:** SARS-CoV-2, Inactivated vaccines, RNA vaccines

## Abstract

Reliable serological assays are needed to understand the real impact of COVID-19. In order to compare the efficiency of different COVID-19 vaccines used in the National Vaccination Program in Tunisia, we have developed a quantitative in-house ELISA. The ELISA is based on the ectodomain of the SARS-CoV-2 Spike Baculovirus recombinant protein. We used a panel of 145 COVID-19 RT-PCR positive serum samples and 116 pre-pandemic serum samples as a negative panel. The validation was carried out by comparison to four commercial techniques (Vidas SARS-CoV-2 IgG anti-RBD Biomérieux, Elecsys Anti-Nucleocapsid of SARS-CoV-2 Roche, cPass GenScript and the quantitative Elecsys Anti-RBD of SARS-CoV-2, Roche). For the evaluation of the National Vaccination campaign, we have included 115 recipients who received one of the approved vaccines. The qualitative performances of the developed ELISA gave 96% sensitivity, 97.5% specificity and 0.968 accuracy. For the evaluation of the different brand of vaccines in recipients not previously infected with SARS-CoV-2, it seems that mRNA vaccine of Pfizer/BioNTech has shown a higher efficacy compared to inactivated virus vaccines. COVID-19 convalescent individuals have generated poor antibody responses. Nevertheless, when they are vaccinated with any brand of the COVID-19 vaccines, many of them mounted an exponential increase of the induced immune responses, qualified as a “hybrid vigor immunity”. Our developed in-house ELISA seems to be very efficient in evaluating the effectiveness of anti-COVID-19 vaccination. Platforms based on mRNA vaccine are better performing than those based on inactivated virus.

## Introduction

The ongoing global pandemic of coronavirus disease 2019 (COVID-19) is caused by the Severe Acute Respiratory Syndrome Coronavirus 2 (SARS-CoV-2), identified for the first time in December 2019 in Wuhan, China. The disease was declared by the World Health Organization (WHO) as a pandemic on the 11th of March 2020^1^. Up to 23 December 2021, more than 276 million cumulative cases and 5.3 million cumulative deaths have been reported^2^. The high burden of the disease triggered a race towards the development and distribution of effective vaccines, to slow the viral transmission, lower the disease severity and reduce the mortality. While typical vaccine development can take up to 10–15 years, COVID-19 vaccines needed less than a year after the identification of COVID-19^3^. To overcome such a challenge, clinical development, manufacturing scale-up and distribution occurred in parallel for all the COVID-19 vaccines. As of 21 December 2021, 137 vaccines are under clinical trials, with 10 in Phase 4 after being approved for emergency use in humans^4^. Different platforms of approved vaccines are currently being administered all over the world, mRNA vaccines (Pfizer-BioNTech, Moderna); recombinant adenovirus vectored vaccines (AstraZeneca, Cansino, Gamaleya, Johnson Pharm) and inactivated vaccines (Sinopharm, Sinovac). Phase 4 clinical trials, also called post-marketing surveillance trials, studies the side effects caused over time that were not seen in earlier trials by a new vaccine, are currently being performed. These trials also study how well the vaccine works over a long period of time.

Although the introduction of Covid-19 vaccines contributed to decrease the burden of the disease in many parts of the world, new cases have increased significantly in many countries in both vaccinated and non-vaccinated populations due to the emergence of new variants^5^. Recent data suggest that mRNA vaccines including Pfizer/BioNTech and Moderna have higher efficacy and protection against COVID-19 infection^5^.

Vaccines against COVID-19 are not reaching many people in developing countries. Less than 1% of people in low-income countries are fully vaccinated, and just 10% in lower-middle-income countries, are fully vaccinated compared with more than half in high-income countries^6^. In Tunisia, from 3 January 2020 to 23 December 2021, there have been 721,797 confirmed cases of COVID-19 and 25,491 deaths, reported to the WHO. As of 23 December 2021, more than 12.6 million vaccine doses have been administered^7^. Up to 20 December 2021, 46.7% of the Tunisian population were fully vaccinated^8^. In Tunisia, all the platforms of COVID-19 vaccines have been used (Pfizer/BioNtech, Moderna, Spoutnik, AstraZeneca, Johnson & Johnson, Sinovac and Sinopharm).

Recent studies indicate that binding and neutralizing SARS-CoV-2 antibodies elicited by natural infection or vaccination persist for more than 6 months although their concentration decreases over time^9^. The passive transfer of neutralizing antibodies and protection are correlated in non-human primates^10^. While such a link has not yet been defined in humans, individuals with high neutralizing antibody titers could well be better protected against SARS-CoV-2. It was reported that neutralizing antibodies are highly correlated with protection due to their ability to block the viruses from entering the host cells^11^. A recent investigation in France reported that ELISA concentrations between 13 and 141 BAU (Binding Antibody Units)/ml provided only 12.4% protection against SARS-CoV-2; concentrations between 141 and 1700 BAU/ml provided 89.3% protection; and concentrations of 1700 BAU/ml and over provided full protection^12^. Therefore, reliable and highly efficient serology assays are urgently needed to evaluate the efficiency of the administered COVID-19 vaccines.

In our investigation, we have set up and validated a quantitative ELISA technique using a Baculovirus recombinant full length SARS-CoV-2 Spike Glycoprotein. This ELISA technique allowed us to assess and compare the efficiency of the different COVID-19 vaccines used in the National Vaccination Program in Tunisia.

## Material and methods

### Patient sampling and ethical issues

**Figure 1:**
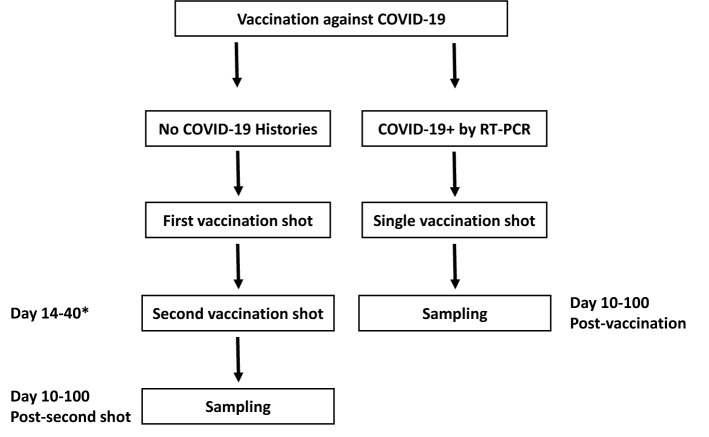
Flowchart for vaccination and sampling procedures. * Second shot, when applicable, was administered according to the manufacturer recommendations as mentioned in “Material and methods”.

The sampling was carried out in the Laboratory of Virology of the “Institut Pasteur de Tunis”, Tunisia. For serotheque constitution, an ethical approval was obtained from the Institutional Review Board of the “Institut Pasteur de Tunis” (Reference: 2020/27/I/LR16IPT). All the samples were collected from patients after obtaining written consent. The selection of samples followed the guidelines of the French “Centre National de Reference des Virus des Infections Respiratoires” published on December 4th 2020, i.e. the evaluation needs at least 50 true positive sera and at least 50 true negative sera. In order to validate our in-house ELISA, we have used a positive and a negative panel. The positive panel was composed of 145 unique, non-duplicated serum samples obtained from COVID-19 confirmed patients based on a positive RT-PCR result from a nasopharyngeal swab. Samples were collected from the first day (D0) until day 109 (D109) after molecular confirmation. Serum samples included 20 sera collected from D1 to D14, 27 sera collected from D15 to D21, 17 sera collected from D22 to D30, 23 sera collected from D31 to D60 and 17 sera collected from D61 to D109. The negative panel was composed of 116 pre-pandemic sera collected before December 2019. They were stored at − 80 °C after collection, then thawed once for this study. For the evaluation of the National Vaccination campaign against COVID-19 in Tunisia, we have recruited 115 recipients of one of the approved vaccines. These recipients voluntarily attended the Laboratory of clinical virology at the “Institut Pasteur of Tunis” to assess their immune responses. All of them were vaccinated at one of the national agreed centers of vaccination. Figure 1 shows a flowchart of sampling and vaccination procedures.

### Recombinant protein

For plate coating of the in-house ELISA we have used the SARS-CoV-2 (2019-nCoV) Spike S1 + S2 ECD-His Recombinant Protein Baculovirus-Insect Cells (Sinobiological, China). It consists of 1209 amino acids and predicts a molecular mass of 134.36 kDa.

### COVID-19 vaccines

For the National Vaccination Program against the COVID-19 in Tunisia following vaccines were administered:

- The mRNA BNT162b2 (Pfizer, Inc., USA and BioNTec, Germany) administered in two shots, 21 days apart into the muscle of the upper arm.
- The mRNA-1273 (Moderna, USA) administered in two shots, 28 days apart into the muscle of the upper arm.
- The Adenovirus Vector JNJ-78436735 (Janssen Pharmaceuticals Companies of Johnson & Johnson, USA) administered in one shot into the muscle of the upper arm.
- The Adenovirus Vector Vaxzevria (previously COVID-19 Vaccine AstraZeneca, UK) is given as two injections, usually into the muscle of the upper arm. The second dose should be given between 4 and 12 weeks after the first dose.
- The Adenovirus Vector Sputnik V (Gamaleya Research Institute of Epidemiology and Microbiology, Russia) administered in two intramuscular shots, by using one type of adenovirus, Ad26, for the first dose, and another, Ad5, for the second.
- The inactivated viruses Coronavax (Sinovac, China) and Sinopharm (Sinopharm, China) used as two doses intramuscularly. WHO recommends an interval of 3–4 weeks between the first and the second dose.

### Enzyme-linked immunosorbent assays

To set up our in-house quantitative ELISA for COVID-19 serology standard techniques were applied. Briefly, 96 well microtiter plates (Nunc) were coated with the SARS-CoV-2 Spike Baculovirus recombinant protein at the concentration of 50 ng/well in 50 µl of 0.1 M carbonate bi-carbonate buffer (pH 9.6). The plates were incubated overnight at 4 °C then saturated with 5% of bovine serum albumin (BSA) and 0.3% sucrose (50 µl/well) in carbonate bicarbonate buffer for 30 min at 37 °C. After washing with PBS containing 0.05% Tween 20 (PBS/T), 50 µl of the serum to be assayed is diluted to 1/50 in PBS/T containing 0.5% BSA and added to appropriate wells. In addition an international standard was serially diluted and deposited in the corresponding wells. The plate was incubated at 37 °C for one hour. The plate was washed with PBS/T and incubated in presence of peroxidase conjugate (anti-Human total IgG, Sigma) at 37 °C for one hour. After washing, 50 µl/well of TMB (Sigma) was added to the wells and the plate incubated for 30 min at room temperature in the dark. The reaction was stopped by addition of 50 µl of HCl (10%) and absorbance measured at 450 nm on a spectrophotometer.

The calibration of our in-house ELISA was carried out against the NIBSC 136/20 (National Institute for Biological Standards and Control, UK), which is the WHO international standard. Its titers are 1300 IU/ml in serum virus neutralization and 476 BAU/ml in anti-Spike IgG ELISA.

For validation of our in-house ELISA we have compared our results to four commercial serological tests all of which are authorized for in vitro diagnosis of SARS-CoV-2 infection and have been approved by the Food and Drug Administration by having an emergency use authorization (FDA-EUA). We have tested the same samples by both the commercial assay and the in-house ELISA following exactly the manufacturer’s procedures.

The first commercial test is Vidas SARS-COV-2 IgG anti-RBD Biomérieux FDA-EUA, which is an automated assay using the ELFA (Enzyme Linked Fluorescent Assay) technique intended for qualitative detection of IgG antibodies to SARS-CoV-2 in human serum or plasma. The second test is Elecsys Anti-Nucleocapsid of SARS-CoV-2 Roche FDA-EUA which is an automated assay based on the ECLIA technique (Electrochemiluminescence) for the qualitative detection of total anti-N antibodies (mainly IgG) to SARS-CoV-2 in human serum and plasma. The third is an ELISA surrogate serum virus neutralization test (cPass GenScriptFDA-EUA); The cPass technology is an ELISA and allows a rapid detection of total virus neutralizing antibodies in a sample by mimicking the interaction between the virus and the host cell receptor ACE2. The final test is Elecsys Anti-RBD of SARS-CoV-2, Roche (quantitative detection of total anti-RBD, FDA-EUA). This test is calibrated against the WHO international standard.

### Statistical analysis

MedCalc V18.2.1 was used for statistical analysis: comparison of two means by t-test, correlation between two quantitative variables by Pearson Correlation, construction of ROC (Receiver Operating Characteristic) curves, determining the analytical performances: sensitivity, specificity, area under the curve (AUC). The *p* values lower than 0.05 are significant at the 95% confidence interval and higher than that are not significant. We have used the USA-Food and Drug Administration (FDA) calculator available on its website to calculate the positive and negative predictive values, on the basis of a prevalence arbitrary fixed by this calculator at 5%. For the calibration accuracy from plate to plate, 100 samples were assayed twice. The means were compared by the paired sample t-test using MedCalc Software. The null hypothesis is that the average of the differences between the paired observations in the two samples is zero. If the calculated *p* value is less than 0.05, the conclusion is that, statistically, the mean difference between the paired observations is significantly different from 0.

## Results

### Validation of an in-house ELISA

**Figure 2:**
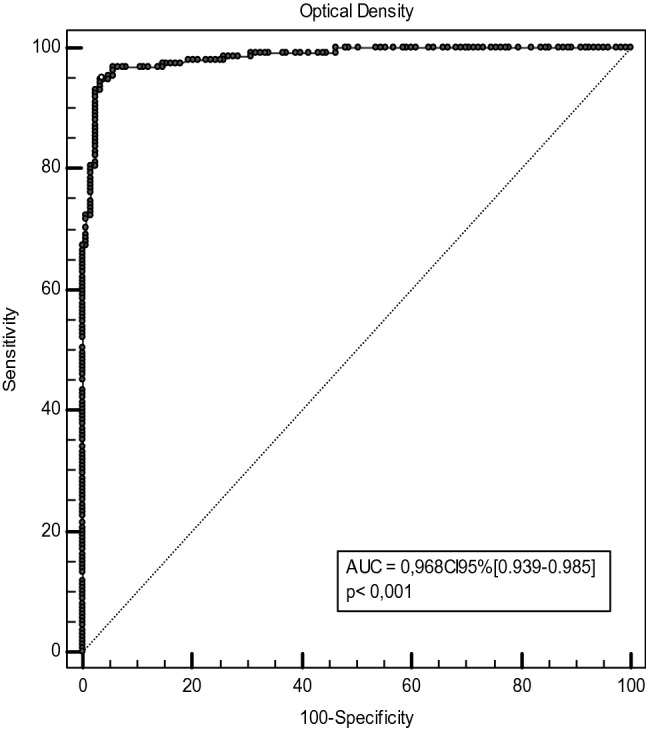
ROC curve for the in-house ELISA using optical density values.

**Figure 3:**
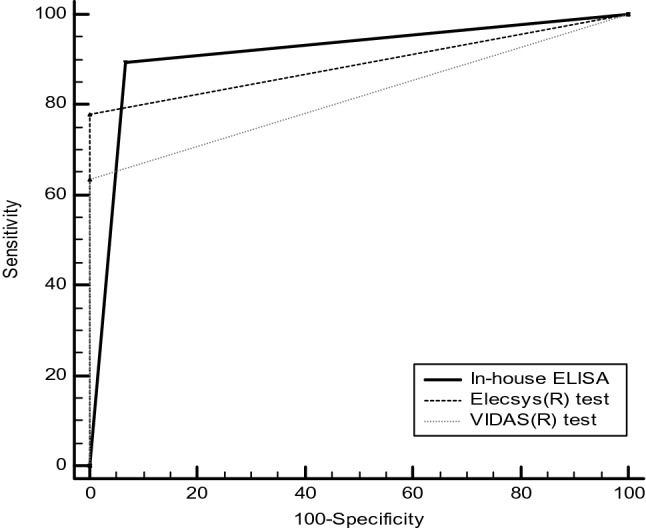
Comparison between the in-house ELISA qualitative results and those of the automated tests Vidas SARS-CoV-2 IgG anti-RBD Biomérieux and Elecsys Anti-Nucleocapsid of SARS-CoV-2 Roche.

We have estimated the qualitative (seropositive or seronegative) performances of our in-house ELISA using several COVID-19 RT-PCR positive samples. ROC analysis (Fig. 2) gave us very satisfactory performances, the overall sensitivity was 96%, CI95% [91.5–98.5%] and the sensitivity after D14 is 95.9%, CI95% [89.8–98.9%] (93 true positive out of 97 patients sampled after D14). For the specificity calculation, we have used 116 pre-pandemic sera collected in 2017 as true negative sera. The specificity of our in-house ELISA was 97.5%, CI95% [92.8–99.5%]. The accuracy of the test was very high 0.968, CI95% [0.939–0.985] and the positive and negative predictive values were 66.7%, CI95% [39.6–85.9%] and 99.8%, CI95% [99.4–99.9%], respectively. The calibration accuracy was guaranteed by using a positive control, a negative control as well as 2 calibrators. In addition, the paired sample t-test gave a *p* value of 0.3562, far higher than 0.05. Therefore, the mean difference between the paired observations is not significantly different from 0 and the accuracy of the given results from plate to plate are robust. The comparison of our qualitative results to those of the Vidas anti-RBD and Elecsys anti-N showed better performances than the former (AUC 0.841, CI95% [0.727–0.883], *p* value < 0.05) but similar performances with the latter (0.885, CI95% [0.808–0.938], *p* value > 0.05) (Fig. 3). Therefore, our developed in-house ELISA is at least as good as the commercial techniques.

**Figure 4:**
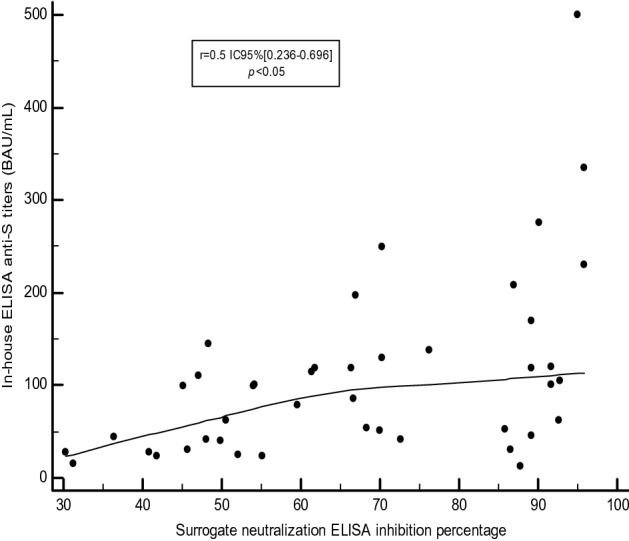
Correlation between the antibody titers (BAU/ml) of the in-house ELISA and the inhibition percentage by the surrogate neutralization ELISA (cPass GenScript).

Next, we compared the quantitative results of our in-house ELISA to those of the commercial cPass GenScript FDA-EUA. Correlation between anti-S titers and percentage of inhibition by the surrogate sero-neutralization ELISA showed a strong correlation (r = 0.5, CI95% [0.236–0.696], *p* value < 0.05) (Fig. 4).

According to the cPass a cut-off of 30% is recommended by the manufacturer as an indicator of the presence of virus neutralizing antibodies. In extrapolation of our in-house test, this corresponds to serums with titers of 59 BAU/ml.

### Evaluation of the humoral immune responses induced by different COVID-19 vaccines

The in-house validated ELISA for COVID-19 serology seems to be efficient in assessing the immune responses induced by the different vaccines used in the National Vaccination Program of Tunisia. We have analyzed 115 recipients of a COVID-19 vaccine and analyzed their immune responses by the quantitative in-house ELISA. Since most of the vaccine platforms are based only on the SARS-CoV-2 Spike and since a history of COVID-19 in vaccine recipients cannot be always confirmed, we have analyzed all the serum samples for anti-N antibodies. Therefore, we have assayed vaccine recipients of either BNT162b2, mRNA-1273, JNJ-78436735, Vaxzevria, or Sputnik V with Elecsys anti-N. With a positive result, we deem the vaccine recipient to have had a previous SARS-CoV-2 infection and when it is negative we assume the recipient has never been infected by the virus. For vaccine recipients of inactivated viruses (Coronavax and Sinopharm), we have shown that the vaccine induced anti-N antibodies are almost undetectable. Therefore, in our analysis when we have high anti-N Elecsys titres of Coronavax and Sinopharm, they are considered previously infected by SARS-CoV-2.

Results are compiled in Table 1, and they are grouped by vaccine brand and sub grouped according to a previous SARS-CoV-2 infection and the number of received shots. In addition, the titers were classified in different intervals and correspond to the percentages of each category of vaccine recipients in each interval. Based on Dimeglio et al*.* report^12^, we have chosen the following intervals of antibody titers. Lower than 10 BAU/ml as seronegative, between 10 and 50 BAU/ml as a poor immune response, between 50 and 200 BAU/ml as a borderline protective immune response, between 200 and 400 BAU/ml as a satisfactory immune response, between 400 and 2500 BAU/ml as a high immune response, between 2500 and 10,000 BAU/ml as a very high immune response, and higher than 10,000 as a hyperimmune response.

**Table 1:** The level of induced humoral immune responses after COVID-19 vaccination in percentages.

Vaccination regimen/level of Ab titer (BAU/ml)	**> 10,000 (%)**	**> 2500 > 10,000 (%)**	**> 400 < 2500 (%)**	**> 200 < 400 (%)**	**> 50 < 200 (%)**	**> 10 < 50 (%)**	**< 10 (%)**
Convalescent non vaccinated (101)	0%	1%	1%	**10.90%**	**42.60%**	**34.70%**	**10.00%**
One shot of BNT162b2 (12)	0%	0%	0%	**16.70%**	**25.00%**	**41.70%**	**16.70%**
Two shots of BNT162b2 (11)	0%	0%	**45.50%**	**18.20%**	**36.40%**	0.00%	0%
COVID-19+/one shot of BNT162b2 (31)	**16.10°%**	**61.30%**	**13.00%**	**6.50%**	0.00%	3.00%	0%
COVID-19+/two shots of BNT162b2 (6)	**16.70°%**	**16.70%**	**16.70%**	0.00%	**50.00%**	0.00%	0%
COVID-19+/one shot of mRNA-1273 (5)		**20.00%**	**80.00%**	0.00%	0.00%	0.00%	0%
One shot of inactivated virus vaccine (6)	0%	0%	0%	**16.70%**	**66.70%**	0.00%	**16.70%**
Two shots of inactivated virus vaccine (12)	0%	0%	0%	0.00%	**41.70%**	**41.70%**	**16.70%**
COVID-19+/one shot of inactivated virus vaccine (5)	0.00%	0.00%	**40.00%**	**40.00%**	**20.00%**	0.00%	0%
COVID-19+/two shots of inactivated virus vaccine (8)	0.00%	0.00%	**50.00%**	**37.50%**	0.00%	**12.50%**	0%
One shot of Vaxzevria (1)	0%%	0%	0%	0.00%	**100.00%**	0.00%	0.00%
Two shots of Vaxzevria (2)	0%%	0%	**100.00%**	0.00%	0.00%	0.00%	0%
COVID-19+/one shot of Vaxzevria (4)	0.00%	**50.00%**	**50.00%**	0.00%	0.00%	0.00%	0%
COVID-19+/two shots of Vaxzevria (1)	0.00%	0.00%	**100.00%**	0.00%	0.00%	0.00%	0%
One shot of JNJ-78436735 (3)	0%	0%	0%	0.00%	**33.30%**	0.00%	**66.70%**
One shot of Sputnik V (1)	0%	0%	0%	0.00%	0.00%	**100.00%**	0.00%
Two shots of Sputnik V (5)	0%	0%	**20.00%**	0.00%	**60.00%**	**20.00%**	0%
COVID-19+/one shot of Sputnik V (2)	0.00%	**50.00%**	0.00%	**50.00%**	0.00%	0.00%	0%

Between parentheses is the number of vaccine recipients in each category.

Significant values are in bold.

In convalescent COVID-19 patients sampled before the beginning of the vaccination campaign, only 1% mounted very or high immune responses in each group. Only 11% of convalescent patients had satisfactory immune responses. The remaining had either induced borderline protective immune responses (42.6%), or a poor response (35%) and some were even seronegative (10%). The mean antibody titers of all convalescent patients was 118.23 BAU/ml.

For recipients of Pfizer/BioNtech, when only one shot is administered and when their anti-N Elecsys test were negative, the induced immune responses were either satisfactory or borderline in 16.7% and 25% respectively. The remaining were either seronegative (16.7%) or mounted a poor immune response (41.7%). Overall, the mean antibody titer was 126 BAU/ml, roughly the same as in convalescent patients. When the second shot was administered, there is still 36.4% with borderline protective responses. The remaining have reached at least satisfactory immune responses with 45.5% categorized as high responders. The mean antibody titer was 532 BAU/ml, significantly higher than convalescent patients (*p* value 0.0004). In the Tunisian COVID-19 vaccination program, whenever a recipient has a previously confirmed infection, they only receive one vaccination. In this category of vaccine recipients, we have also confirmed a previous infection by an anti-N Elecsys positive test. In this category of Pfizer/BioNtech vaccine recipients, hypeimmune (16.1%) and inducer of very high (61.3%) or high (13%) immune responses were detected. Only 6.5% and 3% of them induced satisfactory or poor immune responses, respectively. The mean antibody titer was 7962 BAU/ml, significantly higher than convalescent patients (*p* value 0.000000) and significantly higher than recipients of two doses of Pfizer/Biontech vaccines without prior COVID-19 infection (*p* value 0.043). When people received two shots of Pfizer/BioNtech vaccines normally they should have not been previously infected by the SARS-CoV-2. However, since sometimes there may be lacks of transparency from vaccine recipients, or due to the occurrence of a mild form of the disease, which is not easy to diagnose, they can be administered two shots of Pfizer/BioNtech vaccine. Nevertheless, with the anti-N Elecsys positive test, we have been able to identify these recipients. Although, only six are represented here, half of these individuals have mounted borderline protective immune responses. The remaining have mounted either high, or very high or hyperimmune responses (one recipient in each category). The mean antibody titer was 3064 BAU/ml.

Recipients of Moderna mRNA COVID-19 vaccine are only represented by 5 individuals, all of whom had prior SARS-CoV-2 infection, as confirmed by a positive anti-N test. Their mean antibody titer was 1707 BAU/ml. These individuals received only a single shot of vaccine and have mounted either high (four recipients) or very high (one-recipient) immune responses.

For individuals that have received inactivated virus vaccines we have plotted the results of both brands (Sinovac and Sinopharm). When receiving only one shot of the vaccine, most of them (66.7%) mounted borderline immune responses. The remaining either mounted satisfactory immune responses or they were seronegative, at rates of 16.7% in each category. Their mean antibody titer was 132 BAU/ml very close to that of convalescent patients. When two doses of inactivated virus vaccines were administered, 16.7% of recipients were seronegative. The remaining either mounted poor or borderline immune responses (41.7%, each group). Their mean antibody titer was 49 BAU/ml, not statistically different to that of convalescent patients (*p* value 0.439) and to one shot recipients (*p* value 0.06). In previously infected individuals, based on a positive anti-N serology, inactivated virus vaccine recipients induced almost the same level of immune response, whether they received one, two or three shots. More than 40% of this group induced high immune responses and around 40% had satisfactory antibody titers. Their pooled mean antibody titer was 458 BAU/ml, higher than convalescent patients (*p* value 0.0004) but lower than Pfizer/BioNtech COVID-19 positive recipients after one (*p* value 0.027) or two (*p* value 0.05) shots.

For vaccines based on recombinant adenoviruses, only a few recipients could be recruited to this study. After vaccination with one or two shots of AstraZeneca vaccine, prior SARS-CoV-2 infected individuals mounted at a high level of antibody titers. With Johnson and Johnson vaccine only three recipients were recruited, two of them did not seroconvert and the third only mounted a borderline protective response. Finally, with the Sputnik V vaccine, 60% of non-infected recipients induced a borderline response. Of two previously infected and vaccinated with Sputnik V individuals, one recipient had a very high level of induced immune response. The second recipient induced a satisfactory immune response.

## Discussion

The emergence of viral threats or epidemic is more and more attributable to zoonotic pathogens. Nearly 75% of the newly emerging diseases were linked to an animal origin^13^. Although zoonosis have been thoroughly investigated, they have undergone dramatic changes, due to climate change, human impact on nature, transport of people, animals and food, etc. Consequently, the relationship between man and animal has changed, facilitating the emergence or re-emergence of new zoonosis. Epidemics may threaten all countries, with health, social and economic consequences, as well as a strong media impact. These threats have major consequences on public opinion and not only health professionals must respond very quickly, but also science and researchers should consequently adapt. COVID-19 is a good illustration of this dilemma. The disease was declared as a pandemic by the WHO on the 11th of March 2020^1^ with very high human and economic consequences. Therefore, fighting against the disease is of great importance. For that we need to first, set up platforms for diagnosis and survey and second, apply adequate control measures including the development of efficacious vaccines, whenever that is possible.

Reliable serological assays are needed to understand the real impact of COVID-19 through sero-epidemiological studies, as most cases are asymptomatic and those with mild symptoms are mostly undetected^14^. Testing specific antibody responses to SARS-CoV-2 infection may help diagnosis or in predicting the clinical outcomes^15,16^. Furthermore, serology may predict the level of protection against reinfection or infection after vaccination. Establishing serum virus neutralization techniques for assaying the induced immune responses remain the gold standard. However, neutralization assays are time-consuming and expensive to perform and requires biosafety level 3 laboratory conditions due to the use of pathogenic virus. They are usually not feasible for routine laboratories especially in developing countries. Therefore, specific antibody titers are an alternative to be used as “correlate” or “surrogate” of protection if they correlate with the result of neutralization test. We have developed an in-house ELISA based on the full length of spike ectodomain of SARS-CoV-2 produced by a Baculovirus system. We were able to obtain sensitivity higher than 95% and specificity of 97.5%, very close in performance to what has been reported for other assays^17^. The French recommendations for IgG or total Ig assays at14 days post infection must be greater than 90% and 98%for sensitivity and specificity, respectively^18^. Therefore, our in-house ELISA is valid according to these recommendations. In addition, we have compared the performances of our technique to commercially available techniques (Elecsys and VIDAS) and they were at least as good as those assays. It was previously reported that The Elecsys and the VIDAS methods demonstrated high sensitivities with no false positive results^19^. Furthermore, our in-house developed ELISA showed a good correlation with the quantitative automated anti-RBD test Elecsys. Both were calibrated against the NIBSC 136/20 standard according to the WHO recommendations. Correlations of serology tests with neutralization tests were also demonstrated for many commercial techniques^20,21^. Therefore, our developed in-house ELISA seems to be very efficient in evaluating the effectiveness of anti-COVID-19 vaccination programs.

It is largely accepted that vaccines remain the most effective tool for preventing infectious diseases and improving global health^22^. However, difficulties facing vaccinologists include predicting the type and timing of the next pandemic, developing vaccines to combat rapidly changing pathogens, and establishing rapid-response strategies to control emerging and reemerging infectious diseases. Against COVID-19, developed vaccines have shown acceptable safety records and effectively reduce the death, severity, symptomatic case numbers, and infections resulting from SARS-CoV-2 across the world^23^. In the context of a global pandemic and the continuous emergence of SARS-CoV-2 variants, accelerating vaccination and improving vaccination coverage is still the most important target.

Tunisia started its National Vaccination Program against COVID-19 on the 13th of March 2021. All the WHO authorized vaccine platforms have been used in this program. A proper assessment of the efficiency of the used vaccines has been addressed by serology surveys using our developed in-house ELISA. As far as when humoral immune responses are concerned, and when only vaccine recipients not previously infected with SARS-CoV-2 were considered, it seems that mRNA vaccine of Pfizer/BioNTech has a higher efficacy compared to inactivated virus vaccines, similar to what has been reported elsewhere^5^. For the other surveyed vaccine platforms, the enrolled COVID-19 naïve recipients are rather low and robust interpretations cannot be driven. In a systematic review and meta-analysis of 58 studies, the pooled vaccine effectiveness was 85% (80–91%) for the prevention of Alpha variant of SARS-CoV-2 infections, 75% (71–79%) for the Beta variant, 54% (35–74%) for the Gamma variant, and 74% (62–85%) for the Delta variant^23^. It seems clear that all the current COVID-19 vaccines have a drop of effectiveness against the emerging variants. Even though, Pfizer/BioNtech fully-vaccinated individuals induced at least borderline immune responses, with the drop of titers over time, the level of induced antibodies^24^ and with the emergence of the different variants, it is recommended to administer a third shot of the vaccine^25^. The situation is worse with the inactivated virus vaccines, since they induce lower and quickly declining humoral immune responses^26^. Hence, against variants, virus neutralization tests remain the gold standard for the analysis of the correlation of protection and the efficacy of the vaccine used. Nevertheless, ELISA should first be calibrated against virus neutralization tests using the different circulating variants, then the results can be extrapolated.

We have shown that almost 90% of COVID-19 convalescent individuals have only generated poor or borderline protective antibodies. However, when they are vaccinated with any brand of COVID-19 vaccines, an exponential increase of the induced immune responses is elicited. It was reported that when previously infected individuals are vaccinated an impressive synergy occurs, giving what the authors qualified as a “hybrid vigor immunity”^27,28^. When natural immunity to SARS-CoV-2 is combined to vaccination, greater immune responses were generated. . Hence, we were able to detect very high immune and even hyperimmune responses (titers higher than 10,000 BAU/ml) especially after vaccination with Pfizer/BioNtech mRNA vaccines. It was reported that differences between the memory B cells triggered by infection and those triggered by vaccination could explain the potent hybrid immunity^29,30^. However, vaccination of convalescent individuals did not give systematically high immune responses. In our investigation, 50% of anti-N positive individuals vaccinated with two shots of BNT162b2, only developed borderline protective immune responses. Nevertheless, we should mention that in the National Vaccination Program of Tunisia normally COVID-19 patients receive only one shot of the vaccine. Since they have received two shots that means they have previously developed a milder or a silent form of the disease and they were not aware of being infected. Multiple factors contribute to the degree of immune response mounted following infection. Both binding and neutralizing antibody titers rise faster and reach a higher peak in people with more severe COVID-19^31,32^. Hence, we can speculate that asymptomatic or paucisymptomatic COVID-19 patients do not develop a potent hybrid immunity after being vaccinated.

## Data Availability

The datasets used and/or analyzed during the current study are available from the corresponding author on reasonable request.
